# Supplementary material for: Iterative improvement in the automatic modular design of robot swarms
Source: PeerJ Comput Sci. 2020 Dec 7;6:e322. doi: 10.7717/peerj-cs.322 (PMC7924708; doi:10.7717/peerj-cs.322)
Supplement: Supplemental Information 3 [file peerj-cs-06-322-s003.zip › argos3/doc/api/standalone/a00601_source.html]

ARGoS: plugins/simulator/physics\_engines/dynamics2d/dynamics2d\_box\_model.h Source File


- Main Page
- Related Pages
- Namespaces
- Classes
- Files

- File List
- File Members

# plugins/simulator/physics\_engines/dynamics2d/dynamics2d\_box\_model.h

Go to the documentation of this file.

```
00001 
00007 #ifndef DYNAMICS2D_BOX_MODEL_H
00008 #define DYNAMICS2D_BOX_MODEL_H
00009 
00010 namespace argos {
00011    class CDynamics2DStretchableObjectModel;
00012    class CDynamics2DBoxModel;
00013 }
00014 
00015 #include <argos3/plugins/simulator/physics_engines/dynamics2d/dynamics2d_stretchable_object_model.h>
00016 #include <argos3/plugins/simulator/entities/box_entity.h>
00017 
00018 namespace argos {
00019 
00020    class CDynamics2DBoxModel : public CDynamics2DStretchableObjectModel {
00021 
00022    public:
00023 
00024       CDynamics2DBoxModel(CDynamics2DEngine& c_engine,
00025                           CBoxEntity& c_entity);
00026       virtual ~CDynamics2DBoxModel() {}
00027    };
00028 
00029 }
00030 
00031 #endif
```

---

Generated on 10 Jul 2018 for ARGoS by 
 1.6.1
